# Comparative transcriptomic analysis of deep- and shallow-water barnacle species (Cirripedia, Poecilasmatidae) provides insights into deep-sea adaptation of sessile crustaceans

**DOI:** 10.1186/s12864-020-6642-9

**Published:** 2020-03-17

**Authors:** Zhibin Gan, Jianbo Yuan, Xinming Liu, Dong Dong, Fuhua Li, Xinzheng Li

**Affiliations:** 1 Institute of Oceanology, Chinese Academy of Sciences, Qingdao, 266071 China; 2 Center for Ocean Mega-Science, Chinese Academy of Sciences, Qingdao, 266071 China; 3 University of Chinese Academy of Sciences, Beijing, 100049 China; 4 Laboratory for Marine Biology and Biotechnology, Pilot National Laboratory for Marine Science and Technology (Qingdao), Qingdao, 266237 China

**Keywords:** Barnacle, Deep-sea habitat, Transcriptome, Adaptation

## Abstract

**Background:**

Barnacles are specialized marine organisms that differ from other crustaceans in possession of a calcareous shell, which is attached to submerged surfaces. Barnacles have a wide distribution, mostly in the intertidal zone and shallow waters, but a few species inhabit the deep-sea floor. It is of interest to investigate how such sessile crustaceans became adapted to extreme deep-sea environments. We sequenced the transcriptomes of a deep-sea barnacle, *Glyptelasma gigas* collected at a depth of 731 m from the northern area of the Zhongjiannan Basin, and a shallow-water coordinal relative, *Octolasmis warwicki*. The purpose of this study was to provide genetic resources for investigating adaptation mechanisms of deep-sea barnacles.

**Results:**

Totals of 62,470 and 51,585 unigenes were assembled for *G. gigas* and *O. warwicki*, respectively, and functional annotation of these unigenes was made using public databases. Comparison of the protein-coding genes between the deep- and shallow-water barnacles, and with those of four other shallow-water crustaceans, revealed 26 gene families that had experienced significant expansion in *G. gigas*. Functional annotation showed that these expanded genes were predominately related to DNA repair, signal transduction and carbohydrate metabolism. Base substitution analysis on the 11,611 single-copy orthologs between *G. gigas* and *O. warwicki* indicated that 25 of them were distinctly positive selected in the deep-sea barnacle, including genes related to transcription, DNA repair, ligand binding, ion channels and energy metabolism, potentially indicating their importance for survival of *G. gigas* in the deep-sea environment.

**Conclusions:**

The barnacle *G. gigas* has adopted strategies of expansion of specific gene families and of positive selection of key genes to counteract the negative effects of high hydrostatic pressure, hypoxia, low temperature and food limitation on the deep-sea floor. These expanded gene families and genes under positive selection would tend to enhance the capacities of *G. gigas* for signal transduction, genetic information processing and energy metabolism, and facilitate networks for perceiving and responding physiologically to the environmental conditions in deep-sea habitats. In short, our results provide genomic evidence relating to deep-sea adaptation of *G. gigas*, which provide a basis for further biological studies of sessile crustaceans in the deep sea.

## Background

Conditions on the deep-sea floor are poorly known but generally are considered too harsh for the survival of most organisms, e.g., high hydrostatic pressure, darkness, hypoxia, low temperature, and limited food availability [1–5]. However, a macrofauna consisting of a growing range of newly discovered animals adapted to deep-sea habitats has been reported, including crustaceans [6–8], polychaetes [9, 10], fishes [11, 12], and mollusks [13, 14]. Various mechanisms have adapted them for survival in deep-sea environments: e.g., squat lobsters and mussels have developed chemoautotrophic systems of symbiotic bacteria for inhabiting hydrothermal vents and cold seeps in the seafloor [15–17]; and snailfish have evolved special morphological and physiological characters to survive and thrive in the hadal zone [12]. Studies aimed at understanding survival strategies and adaptive evolution of organisms living in deep seas have also employed genomic or transcriptomic sequencing. For example, in the amphipod *Hirondellea gigas*, adaptation to the hadal environment is associated with gene family expansion and amino acid substitutions of specific proteins [6]; and the shrimp *Rimicaris* sp. upregulates genes associated with sulfur metabolism and detoxification to survive in deep-sea hydrothermal vent environments [8]. However, our understanding of deep-sea adaptation mechanisms remains incomplete, especially for sessile species. Although next-generation sequencing technology is now highly developed, and a few transcriptomic analyses of bio-adhesion mechanisms and development have been reported [18–20]. Merely genetic resources of adult barnacles were surveyed in Cirripedia except for *Pollicipes pollicipes* (https://www.ncbi.nlm.nih.gov/bioproject/PRJNA394196) and *Neolepas marisindica* [21], none relate to adaptive mechanisms in barnacles.

Thoracica barnacles are a unique group of marine crustaceans, enclosed by a mantle and calcareous plates, whose adults are permanently attached to the substrate. Thoracica barnacles are well-known to the public as fouling organisms, adhering to many artificial structures, including vessels and submarine cables, causing structural damage, and increasing fuel consumption. Simultaneously, they are ecologically and economically important species and have been the focus of many studies in developmental biology, crustacean evolution, and ecotoxicology [22–24]. Most barnacles inhabit shallow or tidal marine waters [25, 26] but a few occur in deeper water, even in hadal zones and around hydrothermal vents or cold seeps [27–31]. Stalked barnacles in the family Poecilasmatidae are distributed from the shallow subtidal zone to depths > 3600 m [32]. Within this family, the genus *Glyptelasma* is a typical deep-sea inhabitant with *G. gigas* distributed in the Indo-West Pacific at depths ranging from 236 m to 1092 m. Conveniently, a coordinal shallow-water species, *Octolasmis warwicki*, is distributed in a neighboring region at depths < 100 m [32]. Recently, we successfully collected *G. gigas* and *O. warwicki* individuals at Zhongjiannan Basin and Weizhou Island respectively, and were able to investigate deep-sea adaptation mechanisms through transcriptome sequencing. Zhongjiannan Basin is a Cenozoic sedimentary basin located in the narrowest part of the South China Sea shelf with depths extending down to 4000 m [33, 34]. In the northern area of Zhongjiannan Basin, specific geological structures, including mud volcanoes and pockmarks, are common on the seafloor [35, 36]. Comparing genetic information between these two species could help explain how *G. gigas* has migrated and adapted to this complex environment.

In this study, we sequenced the transcriptomes of the deep-sea barnacle *G. gigas* and a shallow-water barnacle *O. warwicki*. Comparative transcriptomics analysis was performed on them to investigate the genetic changes associated with adaptation to the deep-sea habitat. The main objective of our project was to provide a genomic resource for deep-sea barnacles and to probe the genetic strategies and adaptation of *G. gigas* to the severe conditions of deep-sea environments.

## Results

### Profile of transcriptome assembly and annotation

A total of 120,824,422 and 122,043,504 raw reads were generated from *G. gigas* and *O. warwicki*, respectively (Additional file 1: Table S1). After transcriptome assembly, there were 62,470 unigenes with an N50 length of 1708 bp for *G. gigas*. For *O. warwicki*, there were 51,585 unigenes with an N50 length of 2383 bp (Table 1). The quality of two assemblies was comparable or better than that of *Pollicipes pollicipes* (N50 length of 849 bp) and *Neolepas marisindica* (N50 length of 1596 bp). The two transcriptome assemblies were found to be highly complete including more than 95% of the core genes in the two species. Furthermore, 77.30 and 80.21% of the benchmarking universal single-copy orthologs (BUSCOs) were complete and single-copied in *G. gigas* and *O. warwicki*, respectively (Additional file 2: Table S2).

**Table 1:** Summary of unigene annotation

	*Glyptelasma gigas*	*Octolasmis warwicki*
**Trinity assembly**		
Transcript number	107,419	94,245
Unigene number	62,470	51,585
Total length of unigenes	55,470,568	56,103,552
N50 length of unigenes	1708	2383
Mean length of unigenes	888	1088
**Annotation**		
Nr	19,236 (30.79%)	15,958 (30.93%)
Nt	4110 (6.57%)	4794 (9.29%)
Swiss-Prot	14,000 (22.41%)	12,171 (23.59%)
KEGG Orthology	8413 (13.46%)	6535 (12.66%)
PFAM	19,410 (31.07%)	16,440 (31.86%)
GO	19,576 (31.33%)	16,511 (32.00%)
KOG	8544 (13.67%)	6995 (13.56%)
At least one database	25,672 (41.09%)	21,143 (40.98%)

**Fig. 1:**
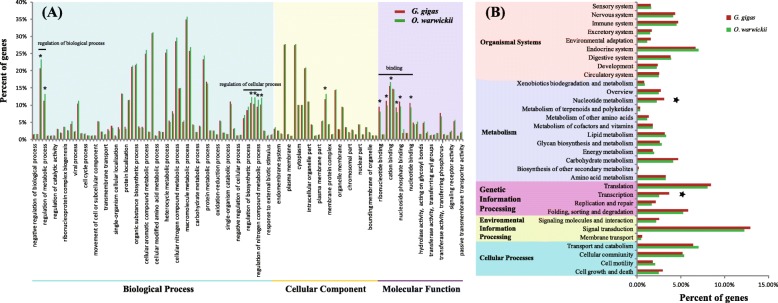
GO (**a**) and KEGG (**b**) distributions of the unigenes of two barnacle species

Among these unigenes, 25,627 (41.09%) and 21,143 (40.98%) were annotated in *G. gigas* and *O. warwicki*, respectively (Table 1). Generally, the distribution of unigenes in GO and KEGG classifications were similar between the two barnacle species (*R*^2^ = 0.9954), suggesting similar genetic structures. The unigenes were mainly assigned to the GO terms: single-organism process (GO:0044699); binding (GO:0005488); organelle (GO:0043226); metabolic process (GO:0008152) (Fig. 1a); and pathways related to signal transduction, translation, and the endocrine system (Fig. 1b). However, some differences were detected between the species. Relatively more unigenes of *O. warwicki* were distributed in the GO terms of regulation of biological process (GO:0050789) and regulation of metabolic process (GO:0019222), whereas in *G. gigas*, relatively more unigenes were distributed in nucleotide binding (GO:0000166) and transferase activity (GO:0016740) (Fisher’s exact test *p* < 0.05). These results were consistent with the findings of KEGG enrichment analysis, which indicated that *G. gigas* had relatively more unigenes with the functions of nucleotide metabolism and transcription (Fig. 1) (Fisher’s exact test *p* < 0.05).

### Phylogenetic tree

**Fig. 2:**
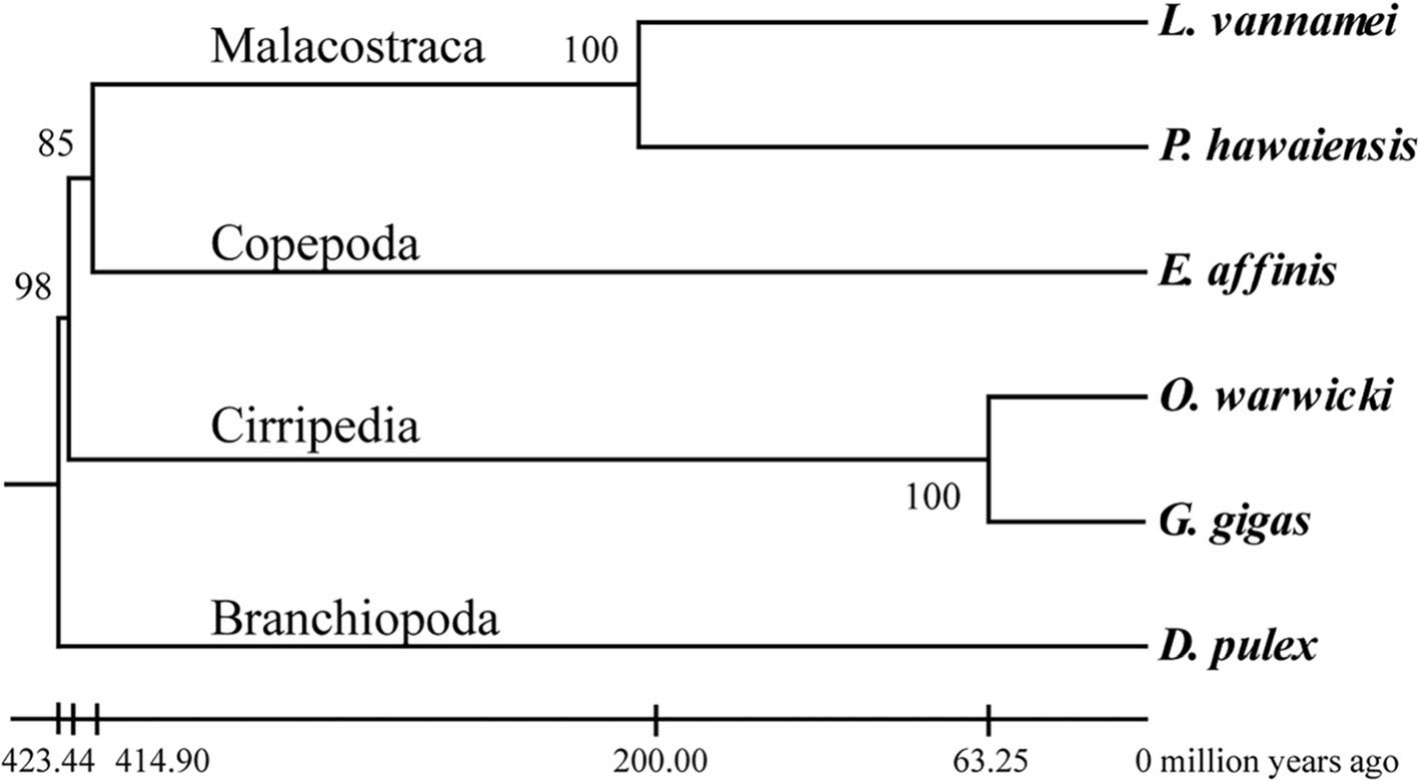
Maximum-likelihood phylogenetic tree based on the single-copy orthologs shared by the both barnacles as well as sequences of Branchiopoda *Daphnia pulex*, copepod *Eurytemora affinis*, and Malacostraca *Parhyale hawaniensis* and *Litopenaeus vannamei*. The estimated divergence times are displayed below the phylogenetic tree

A total of 24,204 gene families were identified in the comparative analysis of the six crustaceans. Among these gene families, all of the protein-coding genes of *G. gigas* and *O. warwicki* were distributed among 10,882 and 9904 gene families, respectively. A set of 566 single-copy gene families (370,657 amino acids) was selected for phylogenetic tree construction. The support values were mostly near 100% on each branch, suggesting high consensus (Fig. 2).

### Gene family expansion

Among the 24,204 gene families, 108 showed distinct expansion in both *G. gigas* and *O. warwicki*. Functional enrichment analysis indicated that these expanded gene families were particularly enriched in pathways of dorso-ventral axis formation, amino-acid biosynthesis and metabolism, and vascular smooth-muscle contraction (Additional file 3: Table S3). This pattern may be related to the unique morphological developmental and life pattern of barnacles, such as well-developed presoma, vestigial abdomen and sessile habit of the adult. Specifically, the major expanded gene family involved broad-complex core protein (Br-C) in the dorso-ventral axis formation pathway. Br-C is required for puffing and transcription of salivary gland late genes during metamorphosis, and is closely related to larval development in insects [37]. In *G. gigas* and *O. warwicki*, 42 and 36 Br-C genes, respectively, were detected. Phylogenetic analysis suggested that these Br-C genes had undergone lineage-specific expansion rather than species-specific expansion, with their distributions nested on the phylogenetic tree (Additional file 7: Fig. S1). Thus, it appears that these clusters of Br-C genes became expanded in the ancestor of *G. gigas* and *O. warwicki* and may have contributed to the adaptive evolution of barnacles.

**Fig. 3:**
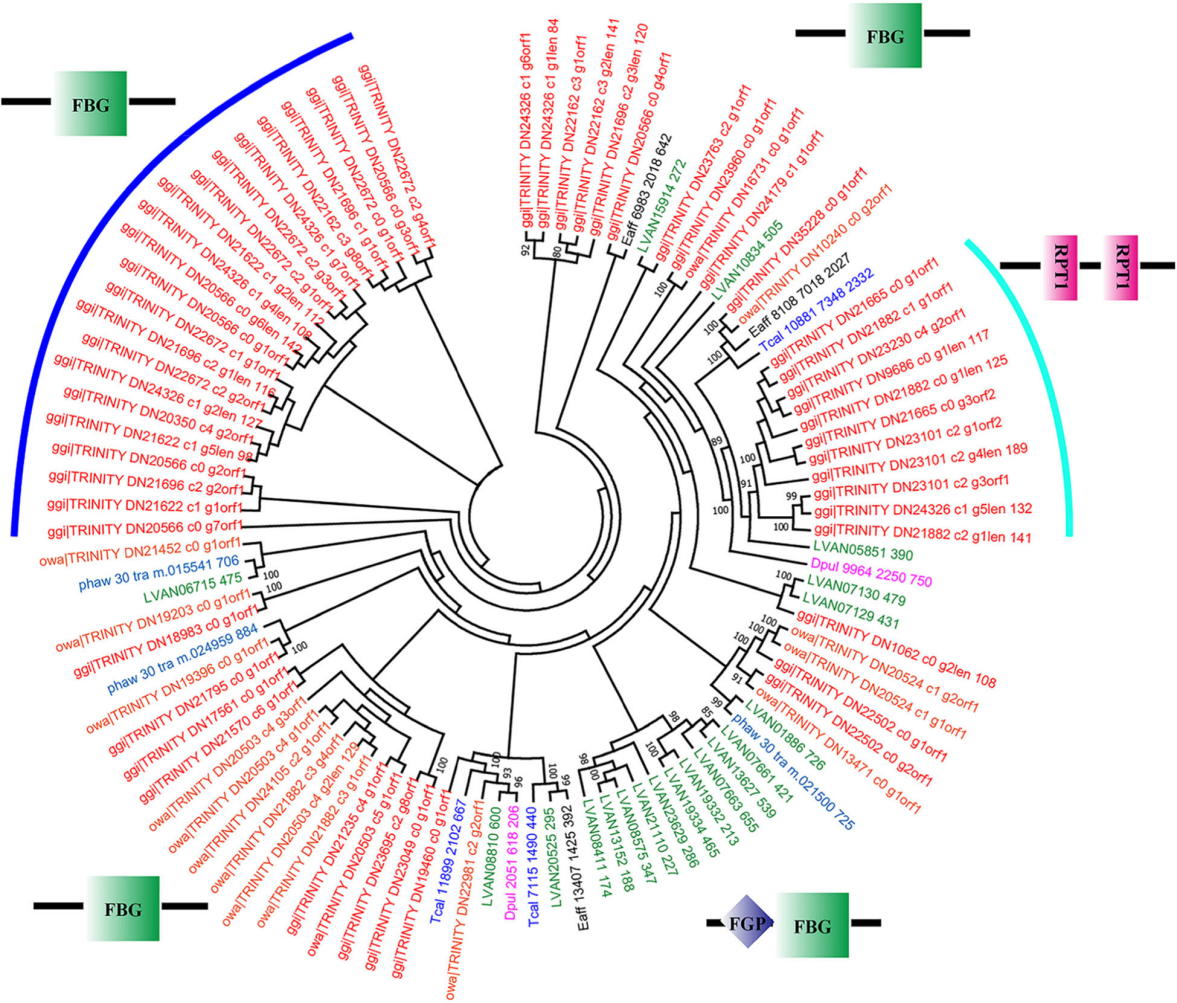
Phylogenetic tree of tenascin gene family. Bootstrap values (> 50%) are shown at branch nodes. ggi: *Glyptelasma gigas*, owa: *Octolasmis warwicki*, Dpul: *Daphnia pulex*, Eaff: *Eurytemora affinis*, LVAN: *Litopenaeus vannamei*, phaw: *Parhyale hawaniensis*

In *G. gigas*, 26 species-specific expanded gene families were identified (Additional file 4: Table S4). These genes were mainly enriched in the pathways of focal adhesion (ko04510), ECM-receptor interaction (ko04512), PI3K-Akt signaling (ko04151), glycosaminoglycan biosynthesis-chondroitin sulfate (ko00532), hippo signaling (ko04391), and axon guidance (ko04360) (Table 2). In our results, tenascin was one of the major expanded gene families which are involved in focal adhesion, ECM-receptor interaction, and the PI3K-Akt signaling pathway. Phylogenetic analysis suggested that tenascin genes in *G. gigas* might have undergone expansion at least twice (Fig. 3). Tenascins are multimeric glycoproteins in the extracellular matrix (ECM) that play key functions in neuronal development, signaling, cell regulation, and axon growth and regeneration [38].

**Table 2:** KEGG pathway annotation of the specifically expanded gene families of *Glyptelasma gigas*

Pathway	DEGs genes with pathway annotation (75)	All genes with pathway annotation (5147)	*P* value	*Q* value	Pathway ID
Focal adhesion	20 (26.67%)	196 (3.81%)	2.01E-12	1.17E-11	ko04510
ECM-receptor interaction	19 (25.33%)	79 (1.53%)	5.14E-19	1.80E-17	ko04512
PI3K-Akt signaling pathway	20 (26.67%)	194 (3.77%)	1.65E-12	1.16E-11	ko04151
Glycosaminoglycan biosynthesis-chondroitin sulfate	6 (8%)	26 (0.51%)	1.43E-06	6.25E-06	ko00532
Hippo signaling pathway	10 (13.33%)	109 (2.12%)	3.14E-06	1.10E-05	ko04391
Axon guidance	6 (8%)	95 (1.85%)	2.43E-03	6.56E-03	ko04360

N-acetylgalactosamine-4-sulfate-6-O-sulfotransferase (CHST15), protocadherin fat 4/16/23 (FAT4/16/23), and plexin are three markedly expanded gene families involved in glycosaminoglycan biosynthesis-chondroitin sulfate, the hippo signaling pathway, and axon guidance, respectively. Furthermore, functional annotation revealed that the species-specific expanded gene families also contained X-ray repair cross-complementing protein 4 (XRCC4), very short patch repair (VSR) endonuclease, the nine-cysteines domain of family 3 (NCD3), and trehalose phosphatase (Table 3).

**Table 3:** Expanded gene families related to KEGG pathway, DNA repair, signal transduction, carbohydrate metabolism in *Glyptelasma gigas*

*G.gigas*	*O.warwicki*	*E.affinis*	*D.pulex*	*P.hawaiensis*	*L.vannamei*	*P* value	Function annotation
focal adhesion, ECM-receptor interaction, and PI3K-Akt signaling pathway							
30	2	0	0	0	0	6.27E-23	Tenascin
hippo signaling pathway-fly							
17	0	0	0	3	2	5.16E-18	Protocadherin fat 4/16/23
axon guidance pathway							
7	1	0	0	1	1	7.53E-03	Plexin-B
DNA repair							
28	8	1	0	1	0	1.40E-04	X-ray repair cross-complementing protein 4 (XRCC4)
20	1	0	0	0	0	2.91E-20	Very short patch repair (VSR) endonuclease
signal transduction							
7	1	0	0	0	0	7.53E-03	Nine Cysteines Domain of family 3 (NCD3)
carbohydrate metabolism							
12	4	0	0	0	0	4.33E-02	Trehalose phosphatase

### Positively selected genes

Genes under positive selection usually respond to natural selection. To identify positively selected genes, we collected 11,611 pairwise best-hit orthologs between *G. gigas* and *O. warwicki*, and performed adaptive evolutionary analyses on them. Our results identified 25 orthologs with ω values > 1.0 (Additional file 5: Table S5) and 118 with ω values > 0.5. These positively selected genes were mainly enriched in the pathways of focal adhesion (ko04510), cyanoamino acid metabolism (ko00460), and RNA transport (ko03013) (Additional file 6: Table S6). Specifically, transcription factor IIA and translation initiation factor eIF-2B were the two positively selected genes involved in RNA transport. Also identified as positively selected were genes encoding excision repair cross-complementation group 4 (ERCC4), calcitonin receptor-like protein, anoctamin-8, G protein-coupled receptor 125 (GPCR 125), discoidin domain receptor 2 (DDR2), WD domain, neurotransmitter-gated ion-channel transmembrane domain, and galactosyltransferase (Table 4).

**Table 4:** Positively selected genes related to KEGG pathway, DNA repair, signal transduction, energy metabolism in *Glyptelasma gigas*

Ortholog	*Glyptelasma gigas*	*Octolasmis warwicki*	ω	Function annotation
RNA transport pathway				
OGB9454	ggi|DN22328_c1_g1	owa|DN23857_c4_g1	1.379	Transcription factor IIA
OGB5776	ggi|DN20749_c0_g1	owa|DN20747_c0_g1	1.225	Translation initiation factor eIF-2B subunit beta
DNA repair				
OGB7222	ggi|DN23236_c0_g1	owa|DN22044_c4_g5	1.232	excision repair cross-complementation group 4 (ERCC4)
signal transduction				
OGB264	ggi|DN12004_c0_g2	owa|DN11255_c0_g1	1.544	calcitonin receptor-like protein, family B
OGB1774	ggi|DN20047_c0_g1	owa|DN15857_c0_g1	1.350	anoctamin-8
OGB10477	ggi|DN15294_c0_g1	owa|DN25773_c0_g1	1.265	G protein-coupled receptor 125 (GPCR 125)
OGB2583	ggi|DN24294_c2_g5	owa|DN17092_c0_g1	1.211	WD domain, G-beta repeat
OGB11272	ggi|DN14199_c0_g1	owa|DN657_c0_g1	1.210	neurotransmitter-gated ion-channel transmembrane domain
energy metabolism				
OGB10876	ggi|DN37033_c0_g1	owa|DN33970_c0_g1	1.641	galactosyltransferase
OGB5160	ggi|DN11355_c0_g1	owa|DN20183_c0_g1	1.220	Discoidin domain receptor 2 (DDR2)

### Differential gene expression between *G. gigas* and *O. warwicki*

**Fig. 4:**
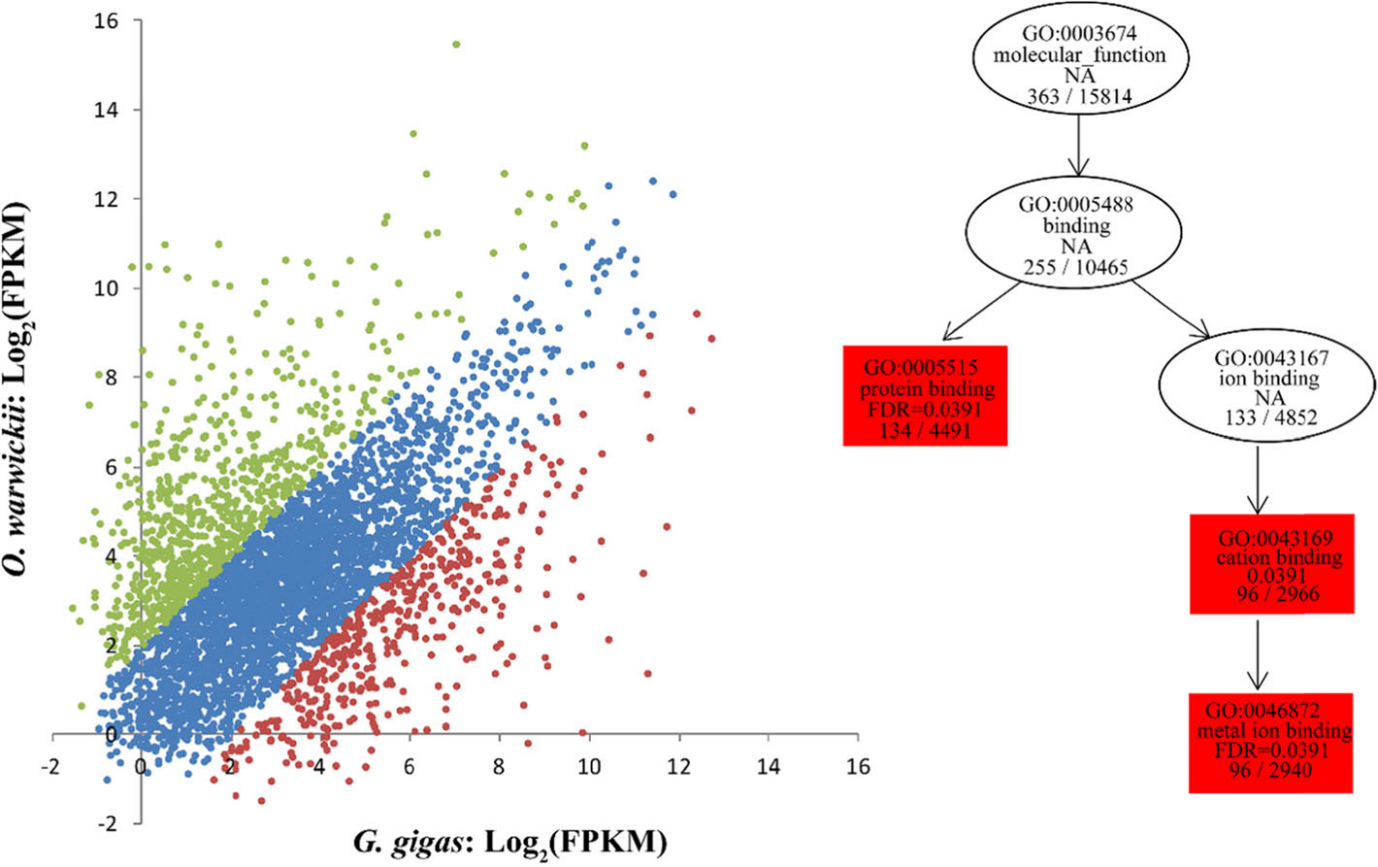
Differentially expressed genes in two barnacle species. The scarter plot in the left is the relative expression level of the orthologs between two barnacle species. The green dots indicated the genes showed highly expression in *O. warwicki*, while the red dots indicated the genes that highly expressed in *Glyptelasma gigas*. The right plot indicated the enriched GO terms of the highly expressed gene in *Glyptelasma gigas*

Generally, orthologous genes between two barnacle species should show similar expression patterns, but there may also be a subset of genes specifically highly expressed in *G. gigas* that were responsible for deep-sea adaptation. Thus, we calculated the relative expression levels (FPKM) of these orthologs to identify the genes that were highly expressed specifically in *G. gigas* and *O. warwicki*. As expected, the expression levels of these orthologs were generally similar between the two barnacle species. However, 480 genes in *G. gigas* and 791 genes in *O. warwicki* showed significantly higher expression levels relatively (Fig. 4). Functional enrichment analysis indicated that the highly expressed genes in *G. gigas* were strongly enriched in the GO terms of binding, especially in metal ion binding (GO:0043167) and protein binding (GO:0005515), and were also enriched in the pathways of galactose metabolism (ko00052), glycosphingolipid biosynthesis (ko00601), glycosaminoglycan biosynthesis (ko map00532), and ubiquitin-mediated proteolysis (ko04120) (*p* < 0.01) (Additional file 8: Fig. S2).

## Discussion

Many potential mechanisms of adaptation to deep-sea environments have been identified. Lan et al. suggested that the expansion of cold-inducible proteins as well as zinc finger domains and positively selected genes related to β-alanine biosynthesis, energy metabolism and genetic information processing played important roles in adaptation to the hadal environment in the amphipod *Hirondellea gigas* [6]. Zhang et al. reported that the expression of the genes associated with sulfur metabolism and detoxification were upregulated in a deep-sea hydrothermal vent shrimp *Rimicaris* sp. [8]. In contrast to those free-swimming species in the deep sea, barnacles are confined to rather narrow zones, which would make adaptation to the deep-sea environment more difficult. In the phylogenetic (Fig. 2), *G. gigas* and *O. warwicki* formed a monophyletic clade that was highly divergent from other crustaceans. The time of divergence of the two barnacle species was estimated to be about 63 million years ago, which is consistent with the divergence of poecilasmatid barnacles [39, 40]. This divergence occurred after the Cretaceous–Palaeogene mass extinction, which was followed by an explosive radiation of organisms [41]. And this time is nearly contemporaneous with the early basement-forming stage of Zhongjiannan Basin [35, 42], suggesting that *G. gigas* was an invasive species in this basin. It has been proposed that deep-water barnacles originated from shallow waters [40, 43], and comparison with shallow-water barnacles might help explain how deep-sea barnacles have adapted to the harsh conditions on the deep-sea floor which characterized by high hydrostatic pressure, darkness, hypoxia, low temperature, and limited food availability.

### Key KEGG pathways implicated in deep-sea adaptation

Our data indicate that focal-adhesion genes were specifically expanded and positively selected in *G. gigas* (Table 2, Additional file 6: Table S6), which suggests that changes in adhesion may have been involved in the adaption of *G. gigas*. For sessile organisms, adhesion is an important process in settlement and survival. In cell biology, focal adhesions are large macromolecular assemblies through which mechanical force and regulatory signals are transmitted between the ECM and an interacting cell. Focal adhesions lead cells to communicate and adhere with their extracellular matrix, and play essential roles in biological processes including cell motility, proliferation, differentiation, gene expression regulation and signal transmission [44]. Other major pathways with specifically expanded genes enriched in *G. gigas* included ECM-receptor interaction, the PI3K-Akt signaling pathway, glycosaminoglycan biosynthesis-chondroitin sulfate, the hippo signaling pathway, and axon guidance (Table 2). Functionally, ECM-receptor interaction, the PI3K-Akt and hippo signaling pathways are three key processes that participate in processing of environmental information [45–47]. Axon guidance represents a key stage in the formation of neuronal networks [48]. Glycosaminoglycan biosynthesis is engaged in glycan metabolic pathways whose products are also mediators of intercellular communication, cellular adhesion, and ECM maintenance [49]. In the meanwhile, cyanoamino acid metabolism and RNA transport were the other two pathways with positively selected genes enriched in *G. gigas* (Additional file 6: Table S6), and they two have roles in amino-acid metabolism and genetic information processing, respectively [50, 51]. Based on these observations, we speculate that *G. gigas* has developed an efficient and synergistic network for environmental perception (ECM-receptor interaction, PI3K-Akt signaling and hippo signaling pathways), Signal transmission (focal adhesion and axon guidance pathways) and physiological response (RNA transport, cyanoamino acid metabolism and glycosaminoglycan biosynthesis pathways). And this network finally results in functional and physiological adjustments that assist *G. gigas* in surviving in the severe and complex deep-sea environments. However, more research is needed to confirm this proposed network.

### Key genes implicated in deep-sea adaptation

Specific genes that were expanded or positively selected in *G. gigas* could facilitate survival in the deep-sea environment. High hydrostatic pressure, hypoxia and low temperature, which characterize the deep-sea floor, could cause DNA damage [52–55] and result in mortality. XRCC4 is one of several break-repair and V(D)J recombination proteins, which could repair DNA double-strand breaks [56]; VSR is an essential component of the very short patch mismatch repair endonucleas, which specifically recognizes and exhibits strand-specific nicking at T-G deoxyribonucleic acid mismatches [57]; and DNA excision repair protein ERCC4 participates in nucleotide excision repair and DNA recombination [58]. In our study, XRCC4 and VSR genes were significantly expanded in *G. gigas* (Table 3), while ERCC4 gene was positively selected (Table 4). All of these genetic processing genes would ensure the structural integrity and normal function of DNA, which might be damaged in the deep-sea environment.

Low temperature and high hydrostatic pressure also lead to the depression of ligand binding and ion channel function in organisms [59–62], which would decrease the efficiency of signal transduction. To counteract the negative effects of low temperature and high hydrostatic pressure on signal transduction, genes encoding ligands and receptors were expanded or positively selected in *G. gigas*. For example, the expanded NCD3 genes encode the nine-cysteines domain of family 3 (Table 3), which is a G protein-coupled receptor (GPCR). The calcitonin receptor-like protein gene and GPCR 125 gene were also positively selected (Table 4). GPCRs constitute a large protein family with essential nodes in signal transduction between the interior and exterior of cells. They bind various ligands, including hormones, neurotransmitters, ions, and other stimuli [63–65]. Genes related to ion channel proteins were also positively selected, e.g., the anoctamin-8 and neurotransmitter-gated ion-channel transmembrane domain (Table 4). The former is a key tether protein that helps Ca^2+^ across membrane transport and assembles all core Ca^2+^-signaling proteins at the endoplasmic reticulum and plasma membrane junctions [66]; the latter is a key domain of ion channels that allows ions, such as Na^+^, K^+^, Ca^2+^, and/or Cl^−^ to pass through the membrane when binding a neurotransmitter [67]. Jointly, the expanded and positively selected of genes concerned with ligand binding and ion channel proteins would help *G. gigas* maintain signal transmission in the deep-sea environment.

Compared with shallow waters, food availability is limited in the deep-sea, which may have encouraged evolution of more efficient energy metabolism [6, 7]. Among the orthologous genes of the two barnacle species, those related to carbohydrate metabolism showed relatively higher expression in *G. gigas* than in *O. warwicki*, including the genes from the pathways of galactose metabolism, glycosphingolipid biosynthesis, and glycosaminoglycan biosynthesis (Fig. 4, Additional file 8: Fig. S2). Accordingly, our results suggested that three key genes that participate in energy metabolic processes, including glycometabolism and lipometabolism, were expanded or positively selected. Trehalose is present in high concentration in insect hemolymph and is consumed during flight [68]. And this sugar has been shown be involved in low-temperature resistance in *Escherichia coli* [69]. Trehalose-phosphatase (Table 3) is used to hydrolyze trehalose-6-phosphate which could be directly converted to glucose and participate in glycolysis. We speculate that trehalose represents a form of energy storage in *G. gigas*, as in insects [70], but more evidence is needed to confirm this conjecture. Galactosyltransferase (Table 4) is a key protein acting in the biosynthesis of disaccharides, oligosaccharides and polysaccharides [71]. DDR2 (Table 4) is a receptor tyrosine kinase activated by collagens, it has diverse functions in cell proliferation, adhesion, migration, extracellular matrix remodeling and reproduction. Remarkably, evidence suggests that it can promote lipid metabolism, although the mechanism is unclear [72]. Corporately, the highly expressed genes, expanded gene families and genes under positive selection involved in energy metabolism may help *G. gigas* use energy efficiently in the harsh conditions of the deep-sea floor.

## Conclusions

The present study is the first to report the transcriptome of a deep-sea barnacle that is compared with that of a shallow-water coordinal species. Our data indicate that *G. gigas* and *O. warwicki* diverged about 63 million years ago, and that *G. gigas* was an invasive species of the Zhongjiannan Basin. By specific gene-family expansion and positive selection of key genes, the deep-sea barnacle *G. gigas* probably evolved an efficient network concerned with environmental perception and physiological response, and acquired adaptive abilities in neural signal transduction, genetic information processing, and energy metabolism. All of these genetic strategies would facilitate confrontation of stress factors and survival in the severe environment of the deep-sea floor. Nevertheless, the present results are preliminary, and the evolutionary mechanisms and precise functional roles of the amplified genes and positively selected genes in genetic adaptation to the deep-sea environment require further confirmation and investigation. This work provides a genomic resource and clues to the genetic adaptation of a deep-sea barnacle that will be helpful for future studies on deep-sea invertebrates.

## Methods

### Sample collection, RNA extraction and sequencing

**Fig. 5:**
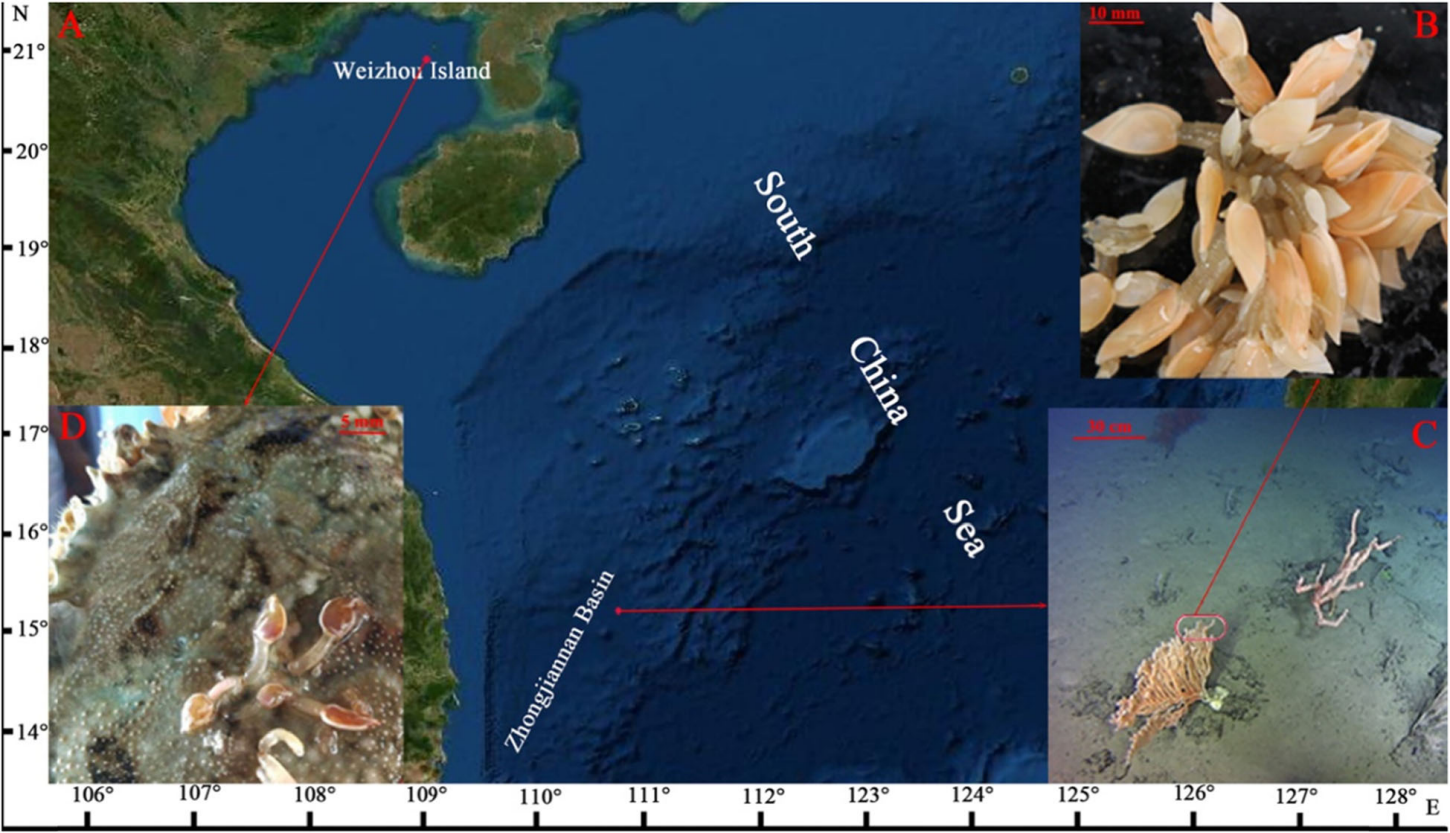
Location of the sampling site and in situ photos of barnacles. **a** Location of the sampling site. **b**, **c** *Glyptelasma gigas* attached to the limb of a gorgonian coral. **d** *Octolasmis warwicki* attached on the carapace of a crab. The base map (**a**) is created by ArcGIS 10 (ESRI, Redlands, CA). Photographs **b** and **c** were taken by the sixth author XZL, and photograph **d** was taken by the third author XML, the map and pictures belong to the authors

Specimens of *G. gigas* were collected from the northern area of Zhongjiannan Basin (15°19.17′N, 110°37.84′E, depth ~ 731 m) in May 2018, during a scientific cruise of the manned submersible *Shenhaiyongshi*. Large numbers of *G. gigas* attached to a limb of a gorgonian coral were acquired (Fig. 5a, b, c). On board, the specimens were immediately frozen in liquid nitrogen and stored at − 80 °C. Specimens of *O. warwicki* were collected from the nearshore waters of Weizhou Island, South China Sea (20°53.95′N, 109°0.61′E, depth ~ 3 m), using a fishing net from a fishing-boat in July 2018. Several specimens of *O. warwicki* attached to the carapace of a crab (Fig. 5a, d) were obtained. After collection, they were immediately immersed in RNAlater solution (Takara, Tokyo, Japan) and stored at − 80 °C.

Three adult individuals randomly selected from each species were pooled to provide sufficient RNA for transcriptome sequencing, a total of six specimens were used for the present research. TRIzol kit (Invitrogen, Carlsbad, CA) was used to extract total RNA following the manufacturer’s instructions. RNA quality was examined by 1% agarose gel electrophoresis, RNA purity was checked using the NanoPhotometer® spectrophotometer (IMPLEN, Westlake Village, CA), RNA concentration was measured using Qubit® RNA Assay Kit in Qubit® 2.0 Fluorimeter (Life Technologies, Carlsbad, CA), and RNA integrity was assessed using the RNA Nano 6000 Assay Kit of the Agilent Bioanalyzer 2100 system (Agilent Technologies, Palo Alto, CA). From each sample, 1.5 μg of RNA was prepared for sequencing. Sequencing libraries were generated using the NEBNext® Ultra™ RNA Library Prep Kit for Illumina® (NEB, Ipswich, MA) following the manufacturer’s instructions and sequenced on an Illumina® HiSeq platform (San Diego, CA). Finally, paired-end reads with length 150 bp were generated. The raw transcriptomic data for *G. gigas* and *O. warwicki* are deposited in NCBI SRA database with accession numbers SRR10523768 and SRR10527303, respectively.

### Transcriptome assembly and annotation

Clean reads were obtained by removal of reads with adaptors and those of low quality from the raw reads (low-quality bases with Qphred ≤5 were > 50% in a read). De novo transcriptome assembly was conducted with Trinity (v2.5.1) using default parameters [73]. The longest transcript of each transcription group was regarded as a unigene for the following analyses. The assembly unigenes of two species were deposited on NCBI TSA database with the accession numbers of GIJX00000000 and GIJW00000000. BUSCO (v1.22) was used to check the quality of the assembly against the database of arthropoda_odb9 [74]. All unigenes were annotated through blasting against public databases, including NCBI non-redundant protein (Nr, E-value 1E-5), Swiss-Prot (E-value 1E-5), and euKaryotic Ortholog Group (KOG, E-value 1E-3) using DIAMOND (v0.8.22) [75], and the NCBI nucleotide database (Nt, E-value 1E-5) using BLAST (v2.2.28+) [76]. Kyoto Encyclopedia of Genes and Genomes (KEGG) classification was performed using the KEGG Automatic Annotation Server (KAAS) with an E-value of 1E-10 [77]. Protein family (Pfam) alignments were carried out using the HMMER (v3.0, http://hmmer.org/) with an E-value of 1E-2, and the Gene Ontology (GO) classification was conducted based on the results of Nr and Pfam using Blast2GO (v2.5) with an E-value of 1E-6 [78]. BLASTx searches were performed for unigenes against Nr database, and followed by conjoining fragmental alignments using SOLAR [79]. Thus, a partial or full open reading frame (ORF) of each unigene was obtained and translated into amino acid sequences.

### Gene family clustering and phylogenetic analysis

Genomic resources for adult barnacles are limited and, therefore, to perform comparative transcriptomic and phylogenetic analyses, the full protein coding genes of four crustaceans, *Daphnia pulex* (PRJNA12756), *Eurytemora affinis* (PRJNA423276); *Parhyale hawaiensis* (PRJNA306836), and *Litopenaeus vannamei* (http://www.shrimpbase.net/lva.download.html) were obtained from NCBI and other databases [80–83]. Pair-wise BLASTp alignment was performed to align all-to-all with an E-value cutoff of 1E-07, and all genes were clustered into gene families using OrthoMCL v2.0.3 [84]. Then, single-copy genes of these species were collected for phylogenetic analysis using maximum-likelihood (ML) methods. Sequence alignment was performed using MUSCLE 3.6 [85]. ML analysis was performed on PhyML with the substitution model WAG + gamma + Inv [86]. One thousand bootstrap replicates were conducted to produce the branch support values. The divergence time was estimated by Bayesian relaxed molecular clock approaches implemented in TIMETREE in MEGA v7.0 [87], with the time calibrations according to the findings of Zhang et al. and Yuan et al. [83, 88]. The expanded and contracted gene families on each branch of the phylogenetic tree were calculated by CAFE [89].

### Identification of positively selected genes

Adaptive evolution was assessed by comparing the nonsynonymous/synonymous substitution ratios (ω = d_N_/d_S_). Orthologs of *G. gigas* and *O. warwicki* were collected by pair-wise best-hit BLAST. Sequence alignment was performed using MUSCLE, and all gaps were removed from the alignment. The ω value of each ortholog was calculated using the program yn00 of PAML v4.48a [90]. Genes with ω > 1.0 were considered fast evolving genes, and ω > 0.5 was considered potential positively selected genes.

### Differential gene expression

Relative levels of gene expression were calculated by mapping clean reads to the assembled unigenes using RSEM [91]. The read counts were calculated using uniquely mapped reads and normalized to the expected number of fragments per kilobase of unigene sequence per million (FKPM). Differential expression analysis was performed on the orthologs of *G. gigas* and *O. warwicki*. Genes with fold change values > 4 were considered to be differentially expressed.

### GO and KEGG enrichment analysis

GO and KEGG enrichment analysis was performed on the expanded gene families, positively selected and differentially expressed genes using Omicshare CloudTools (http://www.omicshare.com/tools/?l=en-us). Enriched GO terms and KEGG Orthology (KO) terms were calculated relative to the background of all unigenes.

## Supplementary information

**Additional file 1:** **Table S1.** Summary of the transcriptome sequencing data.

**Additional file 2:** **Table S2.** BUSCO evaluation of the transcriptome assembly.

**Additional file 3:** **Table S3.** KEGG pathway annotation of expanded gene families both in *Glyptelasma gigas* and *Octolasmis warwicki.*

**Additional file 4:** **Table S4.** Complete list of significantly expanded gene families in *Glyptelasma gigas.*

**Additional file 5:** **Table S5.** Complete list of positively selected genes in *Glyptelasma gigas.*

**Additional file 6:** **Table S6.** KEGG pathway annotation of the positively selected genes of *Glyptelasma gigas.*

**Additional file 7:** **Figure S1.** Phylogenetic tree of Br-C gene family. Bootstrap values (> 50%) are shown at branch nodes. ggi: *Glyptelasma gigas*, owa: *Octolasmis warwicki*, Dpul: *Daphnia pulex*, Eaff: *Eurytemora affinis*, LVAN: *Litopenaeus vannamei*, phaw: *Parhyale hawaniensis.*

**Additional file 8:** **Figure S2.** GO (A) and KEGG (B) distribution of the highly expressed genes in *Glyptelasma gigas.*

## Data Availability

All data presented in this work are provided either in the article or additional files, and the raw transcriptomic data and assembly unigenes for *Glyptelasma gigas* and *Octolasmis warwicki* are deposited in NCBI database (https://www.ncbi.nlm.nih.gov/sra) with accession numbers SRR10523768 and SRR10527303, GIJX00000000 and GIJW00000000, respectively.

## Abbreviations

BUSCOs: Benchmarking universal single-copy orthologs
CHST15: N-acetylgalactosamine-4-sulfate-6-O-sulfotransferase
DDR2: Discoidin domain receptor 2
ECM: Extracellular matrix
ERCC4: Excision repair cross-complementation group 4
FAT: Protocadherin fat
FKPM: Fragments per kilobase of unigene sequence per million
GO: Gene Ontology
GPCR: G protein-coupled receptor
KEGG: Kyoto Encyclopedia of Genes and Genomes
ko: KEGG Orthology
ML: Maximum-likelihood
NCD3: The nine-cysteines domain of family 3
Nr: Non-redundant protein
ORF: Open reading frame
Pfam: Protein family
VSR: Very short patch repair
XRCC4: X-ray repair cross-complementing protein 4
