# Supplementary material for: Comparative transcriptomic analysis of deep- and shallow-water barnacle species (Cirripedia, Poecilasmatidae) provides insights into deep-sea adaptation of sessile crustaceans
Source: BMC Genomics. 2020 Mar 17;21:240. doi: 10.1186/s12864-020-6642-9 (PMC7077169; doi:10.1186/s12864-020-6642-9)
Supplement: Supplementary file 1 — Additional file 1: Table S1. Summary of the transcriptome sequencing data. [file 12864_2020_6642_MOESM1_ESM.pdf]

Additional file 1: Table S1. Summary of the transcriptome sequencing data

Species	Raw Reads	Clean reads	Clean bases	Error(%)	Q20(%)	Q30(%)	GC(%)
Glyptelasma gigas	120824422	116150068	17.42G	0.03	97.23	93.11	55.79
Octolasmis warwicki	122043504	118478528	17.77G	0.03	95.86	90.35	59.71
