# Supplementary material for: Comparative transcriptomic analysis of deep- and shallow-water barnacle species (Cirripedia, Poecilasmatidae) provides insights into deep-sea adaptation of sessile crustaceans
Source: BMC Genomics. 2020 Mar 17;21:240. doi: 10.1186/s12864-020-6642-9 (PMC7077169; doi:10.1186/s12864-020-6642-9)
Supplement: Supplementary file 2 — Additional file 2: Table S2. BUSCO evaluation of the transcriptome assembly. [file 12864_2020_6642_MOESM2_ESM.pdf]

Additional file 2: Table S2. BUSCO evaluation of the transcriptome assembly

	Glyptelasma gigas		*Octolasmis warwicki*	
	Number	Percent	Number	Percent
Complete BUSCOs	971	91.09%	985	92.40%
Complete and single-copy BUSCOs	824	77.30%	855	80.21%
Complete and duplicated BUSCOs	147	13.79%	130	12.20%
Fragmented BUSCOs	44	4.13%	39	3.66%
Missing BUSCOs	51	4.78%	42	3.94%
