# Supplementary material for: Comparative transcriptomic analysis of deep- and shallow-water barnacle species (Cirripedia, Poecilasmatidae) provides insights into deep-sea adaptation of sessile crustaceans
Source: BMC Genomics. 2020 Mar 17;21:240. doi: 10.1186/s12864-020-6642-9 (PMC7077169; doi:10.1186/s12864-020-6642-9)
Supplement: Supplementary file 3 — Additional file 3: Table S3. KEGG pathway annotation of expanded gene families both in Glyptelasma gigas and Octolasmis warwicki. [file 12864_2020_6642_MOESM3_ESM.pdf]

Additional file 3: Table S3. KEGG pathway annotation of expanded gene families both in *Glyptelasma gigas* and *Octolasmis warwicki*

Pathway	DEGs genes with pathway annotation (66)	All genes with pathway annotation (5147)	Pvalue	Qvalue	Pathway ID
Dorso-ventral axis formation	15 (22.73%)	75 (1.46%)	9.86E-15	4.93E-13	ko04320
Arginine and proline metabolism	5 (7.58%)	51 (0.99%)	4.43E-04	1.11E-02	ko00330
Ribosome	11 (16.67%)	322 (6.26%)	2.33E-03	3.41E-02	ko03010
Gastric acid secretion	5 (7.58%)	80 (1.55%)	2.91E-03	3.41E-02	ko04971
Nicotinate and nicotinamide metabolism	3 (4.55%)	26 (0.51%)	3.41E-03	3.41E-02	ko00760
Hematopoietic cell lineage	2 (3.03%)	10 (0.19%)	4.24E-03	3.53E-02	ko04640
Biosynthesis of amino acids	5 (7.58%)	102 (1.98%)	6.82E-03	4.43E-02	ko01230
Platelet activation	5 (7.58%)	106 (2.06%)	7.09E-03	4.43E-02	ko04611
Phagosome	6 (9.09%)	149 (2.89%)	9.52E-03	4.66E-02	ko04145
Vascular smooth muscle contraction	5 (7.58%)	108 (2.1%)	1.11E-02	4.66E-02	ko04270
