# Supplementary material for: Comparative transcriptomic analysis of deep- and shallow-water barnacle species (Cirripedia, Poecilasmatidae) provides insights into deep-sea adaptation of sessile crustaceans
Source: BMC Genomics. 2020 Mar 17;21:240. doi: 10.1186/s12864-020-6642-9 (PMC7077169; doi:10.1186/s12864-020-6642-9)
Supplement: Supplementary file 4 — Additional file 4: Table S4. Complete list of significantly expanded gene families in Glyptelasma gigas. [file 12864_2020_6642_MOESM4_ESM.pdf]

Additional file 4: Table S4. Complete list of significantly expanded gene families in *Glyptelasma gigas*

Glyptelasma gigas	*Octolasmis warwicki*	*Eurytemora affinis*	*Daphnia pulex*	*Parhyale hawaiensis*	*Litopenaeus vannamei*	P value	Function annotation
28	8	1	0	1	0	1.40E-04	X-ray repair cross-complementing protein 4 (XRCC4)
19	8	3	4	1	0	3.51E-02	PREDICTED: uncharacterized protein LOC100568550
30	2	0	0	0	0	6.27E-23	Tenascin
26	1	0	1	1	0	2.19E-34	Titin
20	8	0	0	0	0	2.14E-02	Circovirus capsid protein
17	6	0	0	1	1	1.76E-02	Ribosomal protein L13e
17	0	0	0	3	2	5.16E-18	Protocadherin fat 4/16/23
20	1	0	0	0	0	2.91E-20	Very short patch repair endonuclease (VSR)
17	3	0	0	0	0	4.69E-05	Pro-kumamolisin, activation domain
15	4	0	0	1	0	4.90E-03	Integrase core domain
15	5	0	0	0	0	2.01E-02	hypothetical protein HELRODRAFT_163242
15	4	0	0	0	0	4.90E-03	PREDICTED: uncharacterized protein LOC105338248
14	5	0	0	0	0	3.72E-02	RNA-directed DNA polymerase from mobile element jockey [*Larimichthys crocea*]
10	2	2	0	4	0	6.71E-03	PREDICTED: RNA-directed DNA polymerase from mobile element jockey-like
14	4	0	0	0	0	1.07E-02	PREDICTED: uncharacterized protein LOC105564550
14	1	0	0	1	0	5.28E-10	RNA-directed DNA polymerase from mobile element jockey [*Drosophila melanogaster*]
12	4	0	0	0	0	4.33E-02	Trehalose phosphatase
11	1	0	3	0	0	2.60E-06	hypothetical protein [*Cotesia congregata*]
11	0	1	0	0	2	2.16E-10	PREDICTED: titin [*Tribolium castaneum*]

9	1	0	4	0	0	2.25E-04	hypothetical protein DAPPUDRAFT_118411
9	3	0	0	0	0	9.56E-02	--
9	2	0	0	0	0	1.90E-02	hypothetical protein N307_01365, partial
8	1	0	0	1	0	1.46E-03	--
7	1	0	0	1	1	7.53E-03	Plexin-B [*Drosophila melanogaster*]
8	1	0	0	0	0	1.46E-03	THAP domain
7	1	0	0	0	0	7.53E-03	Nine Cysteines Domain of family 3 GPCR
