# Supplementary material for: Comparative transcriptomic analysis of deep- and shallow-water barnacle species (Cirripedia, Poecilasmatidae) provides insights into deep-sea adaptation of sessile crustaceans
Source: BMC Genomics. 2020 Mar 17;21:240. doi: 10.1186/s12864-020-6642-9 (PMC7077169; doi:10.1186/s12864-020-6642-9)
Supplement: Supplementary file 5 — Additional file 5: Table S5. Complete list of positively selected genes in Glyptelasma gigas. [file 12864_2020_6642_MOESM5_ESM.pdf]

**Additional file 5: Table S5.** Complete list of positively selected genes in *Glyptelasma gigas*

| Ortholog | *Glyptelasma gigas* | *Octolasmis warwicki* | ω | Function annotation |
|---|---|---|---|---|
| OGB5059 | ggi\|DN22954_c3_g2 | owa\|DN20087_c1_g1 | 99.000 | PREDICTED: uncharacterized protein LOC105570728 [*Vollenhovia emeryi*] |
| OGB10732 | ggi\|DN24720_c0_g1 | owa\|DN31130_c0_g1 | 78.374 | Melaminivora sp. SC2-7 chromosome, complete genome |
| OGB8720 | ggi\|DN17814_c0_g1 | owa\|DN23277_c0_g1 | 4.044 | hypothetical protein X975_22095, partial [*Stegodyphus mimosarum*] |
| OGB10941 | ggi\|DN47221_c0_g1 | owa\|DN35020_c0_g1 | 3.141 | hypothetical protein HHA_455820 [*Hammondia hammondi*] |
| OGB10866 | ggi\|DN32046_c0_g1 | owa\|DN33886_c0_g1 | 2.380 | PREDICTED: uncharacterized protein LOC103516194 [*Diaphorina citri*] |
| OGB476 | ggi\|DN19235_c0_g1 | owa\|DN12669_c0_g1 | 1.713 | hypothetical protein CLF_113308 [*Clonorchis sinensis*] |
| OGB6733 | ggi\|DN17149_c0_g1 | owa\|DN21618_c0_g1 | 1.642 | Golgi phosphoprotein 3 (GPP34) |
| OGB10876 | ggi\|DN37033_c0_g1 | owa\|DN33970_c0_g1 | 1.641 | Galactosyltransferase |
| OGB11249 | ggi\|DN26253_c0_g1 | owa\|DN6220_c0_g1 | 1.605 | Echinoderm microtubule-associated protein-like 1/2 |
| OGB264 | ggi\|DN12004_c0_g2 | owa\|DN11255_c0_g1 | 1.544 | Calcitonin receptor-like protein, family B [*Daphnia pulex*] |
| OGB10998 | ggi\|DN12056_c0_g1 | owa\|DN36340_c0_g1 | 1.420 | PREDICTED: E3 ubiquitin-protein ligase RGLG1-like |
| OGB9454 | ggi\|DN22328_c1_g1 | owa\|DN23857_c4_g1 | 1.379 | PREDICTED: homeobox protein PKNOX2-like isoform X2 [*Orussus abietinus*] |
| OGB2513 | ggi\|DN21587_c2_g1 | owa\|DN16991_c0_g3 | 1.351 | -- |
| OGB1774 | ggi\|DN20047_c0_g1 | owa\|DN15857_c0_g1 | 1.350 | PREDICTED: anoctamin-8 isoform X3 [*Acromyrmex echinatior*] |
| OGB3176 | ggi\|DN4943_c0_g1 | owa\|DN17926_c0_g1 | 1.279 | GE16240 [*Drosophila yakuba*] |
| OGB10477 | ggi\|DN15294_c0_g1 | owa\|DN25773_c0_g1 | 1.265 | G protein-coupled receptor 125 |
| OGB7222 | ggi\|DN23236_c0_g1 | owa\|DN22044_c4_g5 | 1.232 | DNA excision repair protein ERCC-4 |
| OGB5776 | ggi\|DN20749_c0_g1 | owa\|DN20747_c0_g1 | 1.225 | Translation initiation factor eIF-2B subunit beta |
| OGB5160 | ggi\|DN11355_c0_g1 | owa\|DN20183_c0_g1 | 1.220 | Giscoidin domain receptor family member 2 |
| OGB3129 | ggi\|DN31318_c0_g1 | owa\|DN17865_c1_g4 | 1.218 | -- |
| OGB2583 | ggi\|DN24294_c2_g5 | owa\|DN17092_c0_g1 | 1.211 | hypothetical protein BRAFLDRAFT_125598 [*Branchiostoma floridae*] |
| OGB11272 | ggi\|DN14199_c0_g1 | owa\|DN657_c0_g1 | 1.210 | Glutamate receptor |
| OGB109 | ggi\|DN3545_c0_g1 | owa\|DN10623_c0_g3 | 1.117 | PREDICTED: uncharacterized protein LOC105442062 |

| | | | | |
|---|---|---|---|---|
| OGB5882 | ggi\|DN13021_c0_g1 | owa\|DN20842_c4_g1 | 1.065 | PREDICTED: guanylate cyclase 32E-like [*Athalia rosae*] |
| OGB4110 | ggi\|DN17588_c0_g1 | owa\|DN19115_c0_g1 | 1.029 | BTB/POZ domain-containing protein 9 [*Zootermopsis nevadensis*] |
