# Supplementary material for: Comparative transcriptomic analysis of deep- and shallow-water barnacle species (Cirripedia, Poecilasmatidae) provides insights into deep-sea adaptation of sessile crustaceans
Source: BMC Genomics. 2020 Mar 17;21:240. doi: 10.1186/s12864-020-6642-9 (PMC7077169; doi:10.1186/s12864-020-6642-9)
Supplement: Supplementary file 6 — Additional file 6: Table S6. KEGG pathway annotation of the positively selected genes of Glyptelasma gigas. [file 12864_2020_6642_MOESM6_ESM.pdf]

Additional file 6: Table S6. KEGG pathway annotation of the positively selected genes of *Glyptelasma gigas*

Pathway	DEGs genes with pathway annotation (75)	All genes with pathway annotation (5147)	Pvalue	Qvalue	Pathway ID
Focal adhesion	3 (13.04%)	94 (3.02%)	3.05E-2	3.67E-1	ko04510
Cyanoamino acid metabolism	1 (4.35%)	5 (0.16%)	3.64 E-2	3.67E-1	ko00460
RNA transport	3 (13.04%)	102 (3.28%)	3.76 E-2	3.67E-1	ko03013
