# Supplementary material for: Comparative transcriptomic analysis of deep- and shallow-water barnacle species (Cirripedia, Poecilasmatidae) provides insights into deep-sea adaptation of sessile crustaceans
Source: BMC Genomics. 2020 Mar 17;21:240. doi: 10.1186/s12864-020-6642-9 (PMC7077169; doi:10.1186/s12864-020-6642-9)
Supplement: Supplementary file 7 — Additional file 7: Figure S1. Phylogenetic tree of Br-C gene family. Bootstrap values (> 50%) are shown at branch nodes. ggi: Glyptelasma gigas, owa: Octolasmis warwicki, Dpul: Daphnia pulex, Eaff: Eurytemora affinis, LVAN: Litopenaeus vannamei, phaw: Parhyale hawaniensis. [file 12864_2020_6642_MOESM7_ESM.pdf]

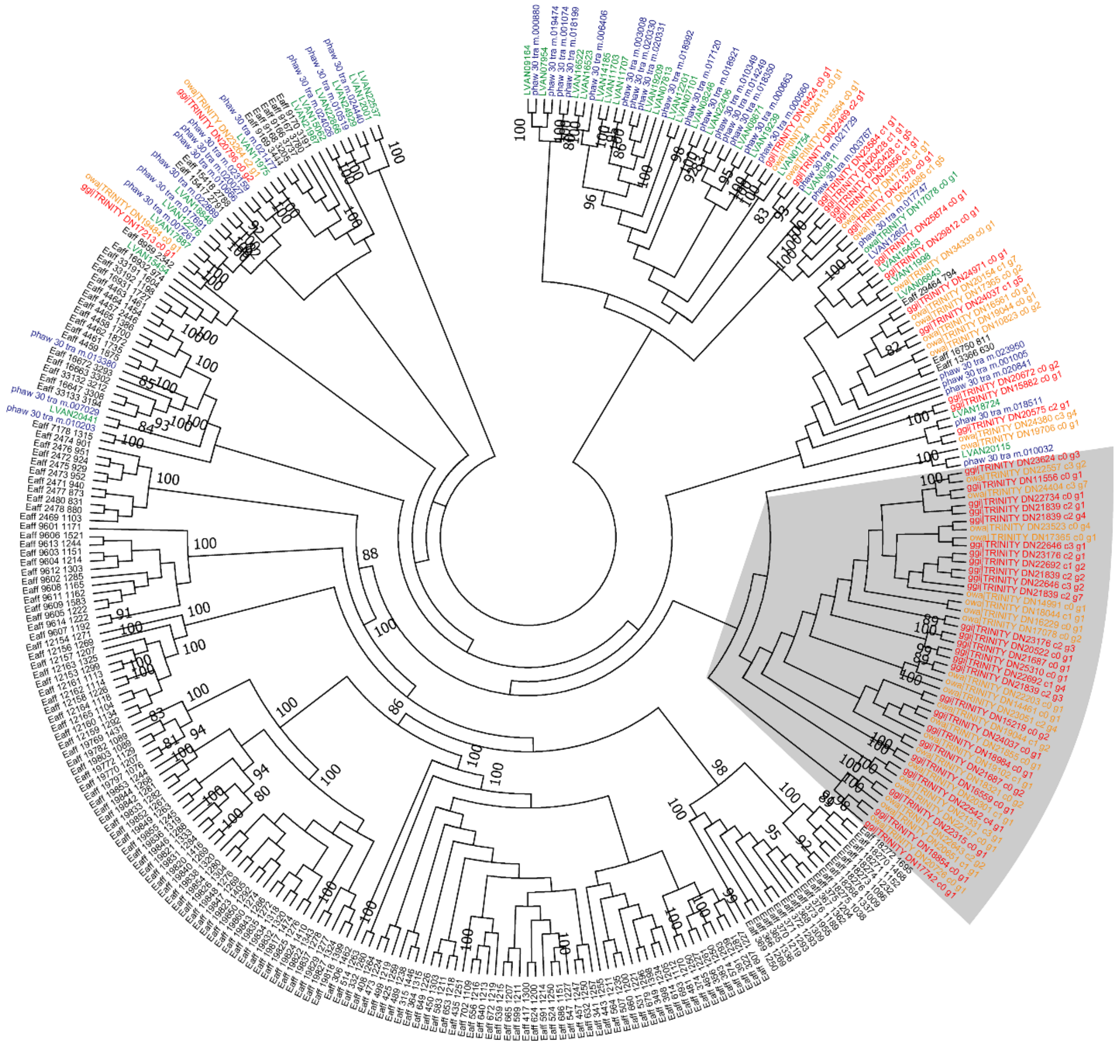

Additional file 7: Figure S1. Phylogenetic tree of Br-C gene family. Bootstrap values (>50%) are shown at branch nodes. ggi: *Glyptelasma gigas*, owa: *Octolasmis warwicki*, Dpul: *Daphnia pulex*, Eaff: *Eurytemora affinis*, LVAN: *Litopenaeus vannamei*, phaw: *Parhyale hawaniensis*
