# Supplementary material for: Comparative transcriptomic analysis of deep- and shallow-water barnacle species (Cirripedia, Poecilasmatidae) provides insights into deep-sea adaptation of sessile crustaceans
Source: BMC Genomics. 2020 Mar 17;21:240. doi: 10.1186/s12864-020-6642-9 (PMC7077169; doi:10.1186/s12864-020-6642-9)
Supplement: Supplementary file 8 — Additional file 8: Figure S2. GO (A) and KEGG (B) distribution of the highly expressed genes in Glyptelasma gigas. [file 12864_2020_6642_MOESM8_ESM.pdf]

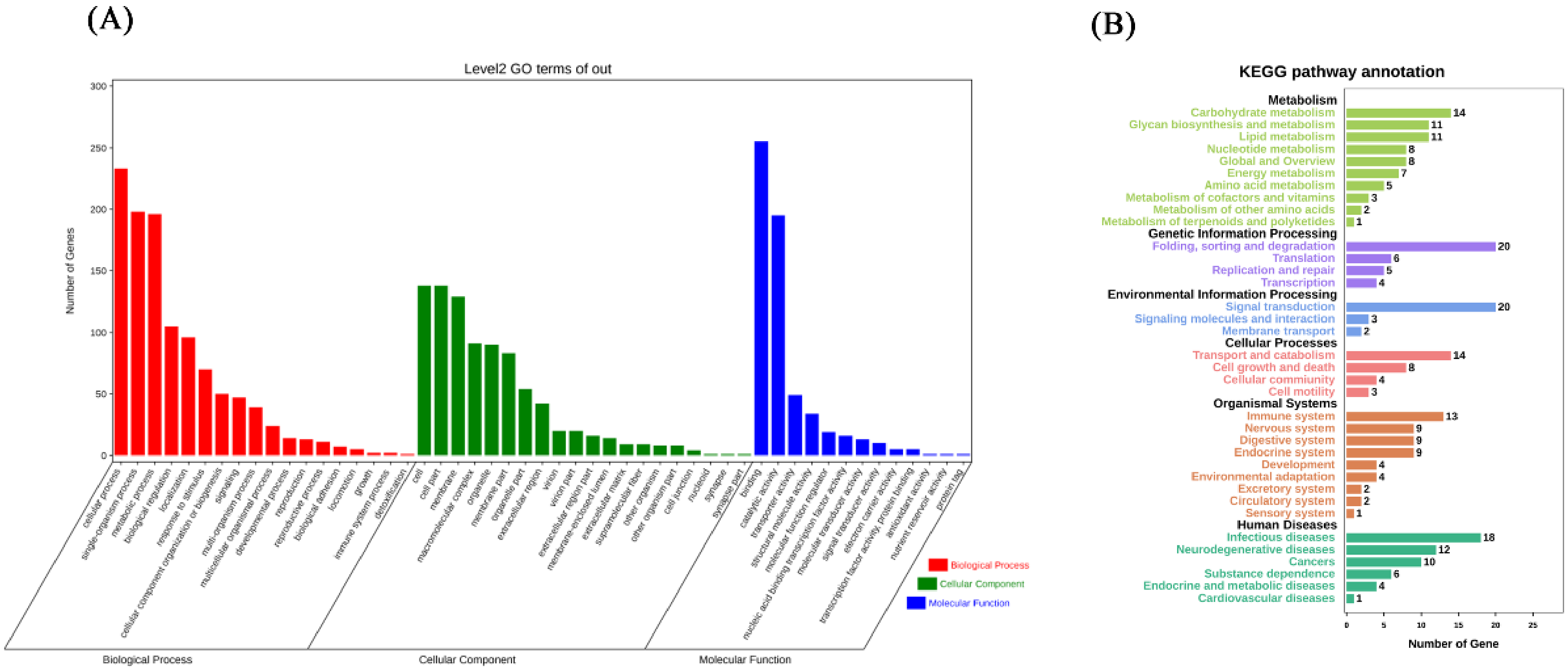

Additional file 8: Figure S2. GO (a) and KEGG (b) distribution of the highly expressed genes in *Glyptelasma gigas*
